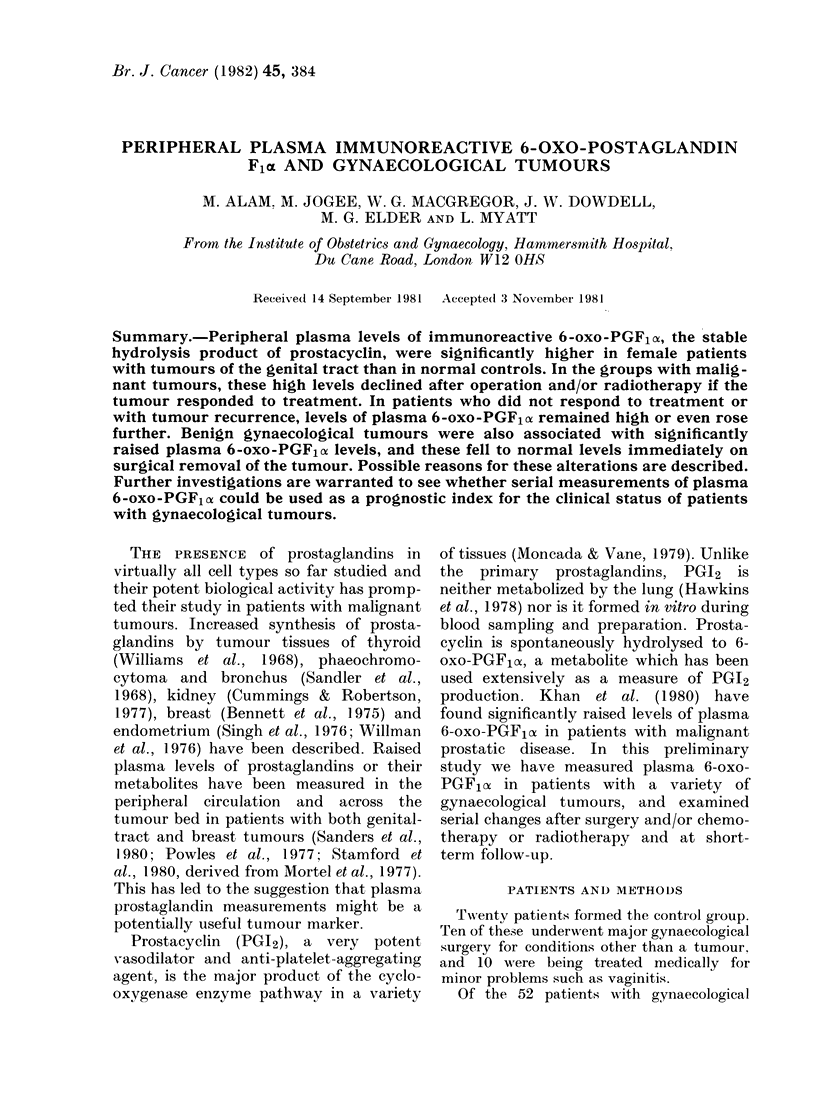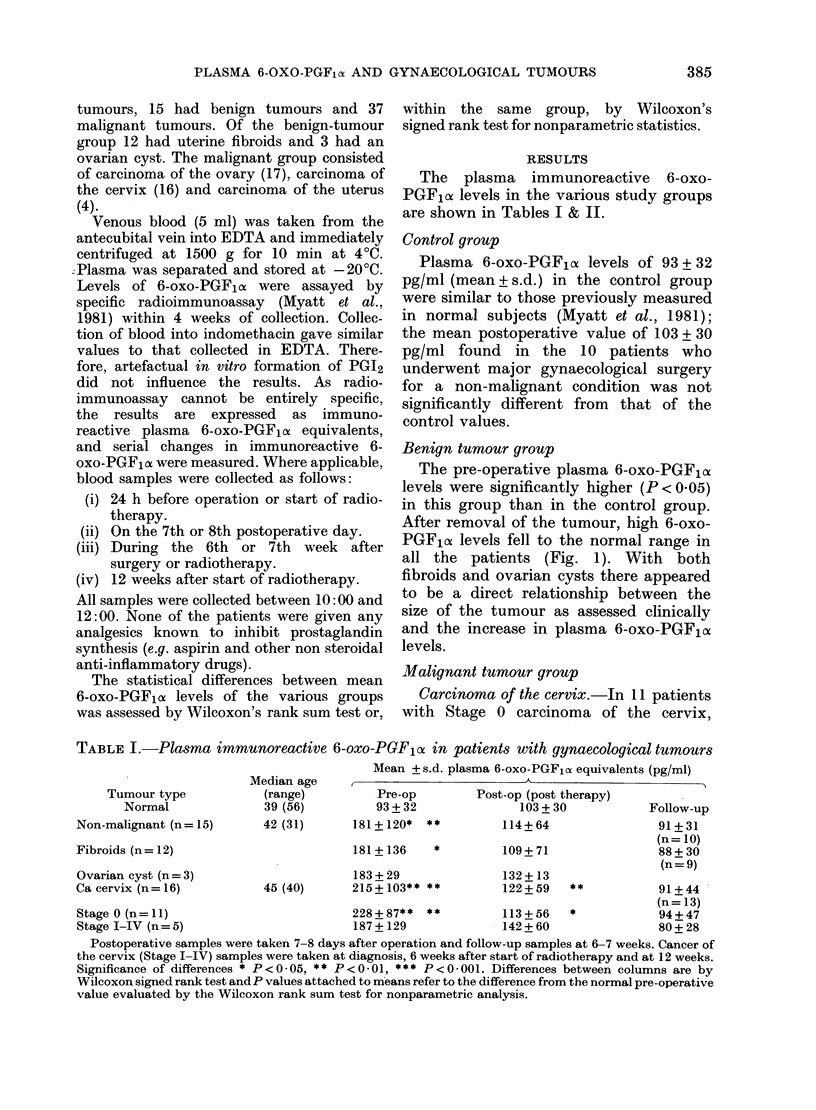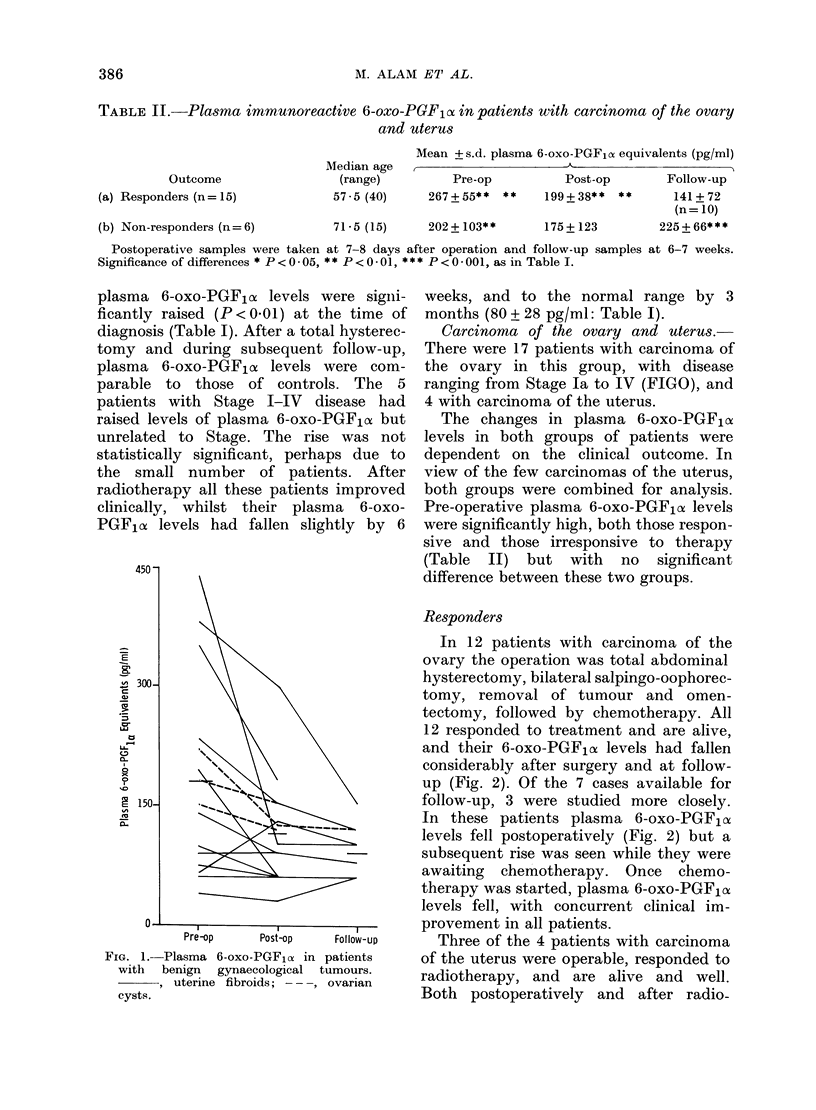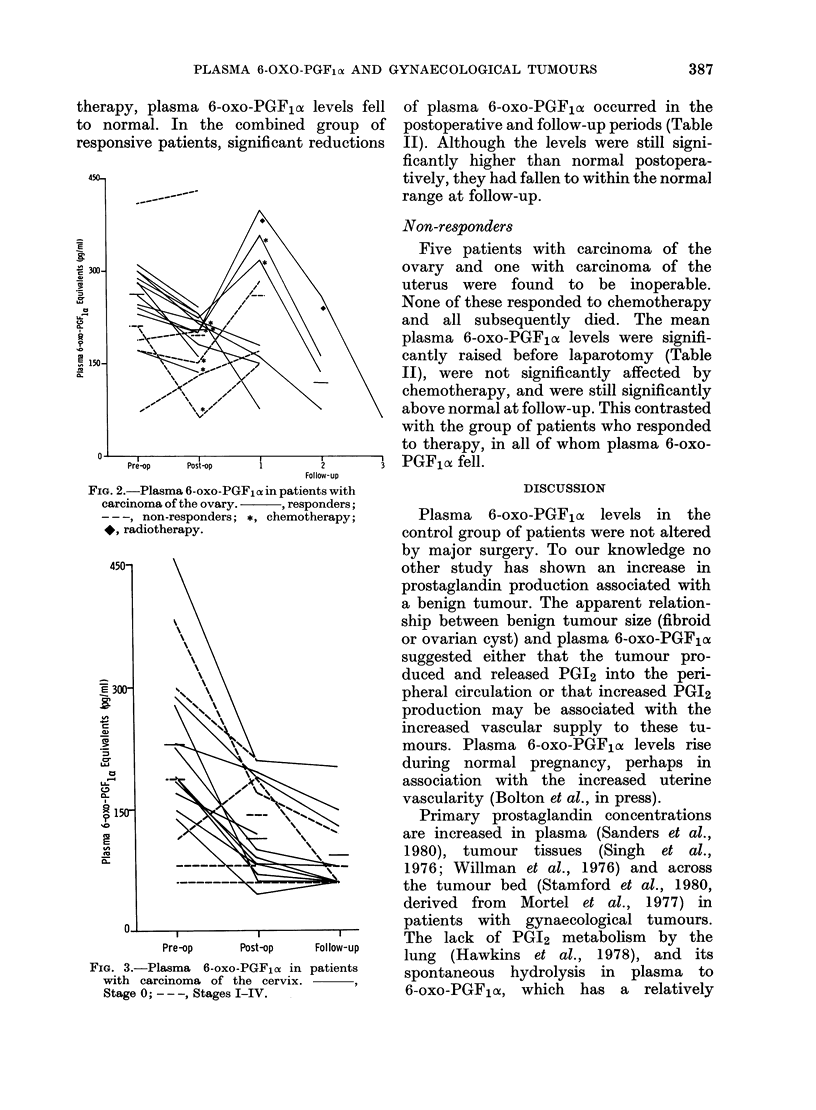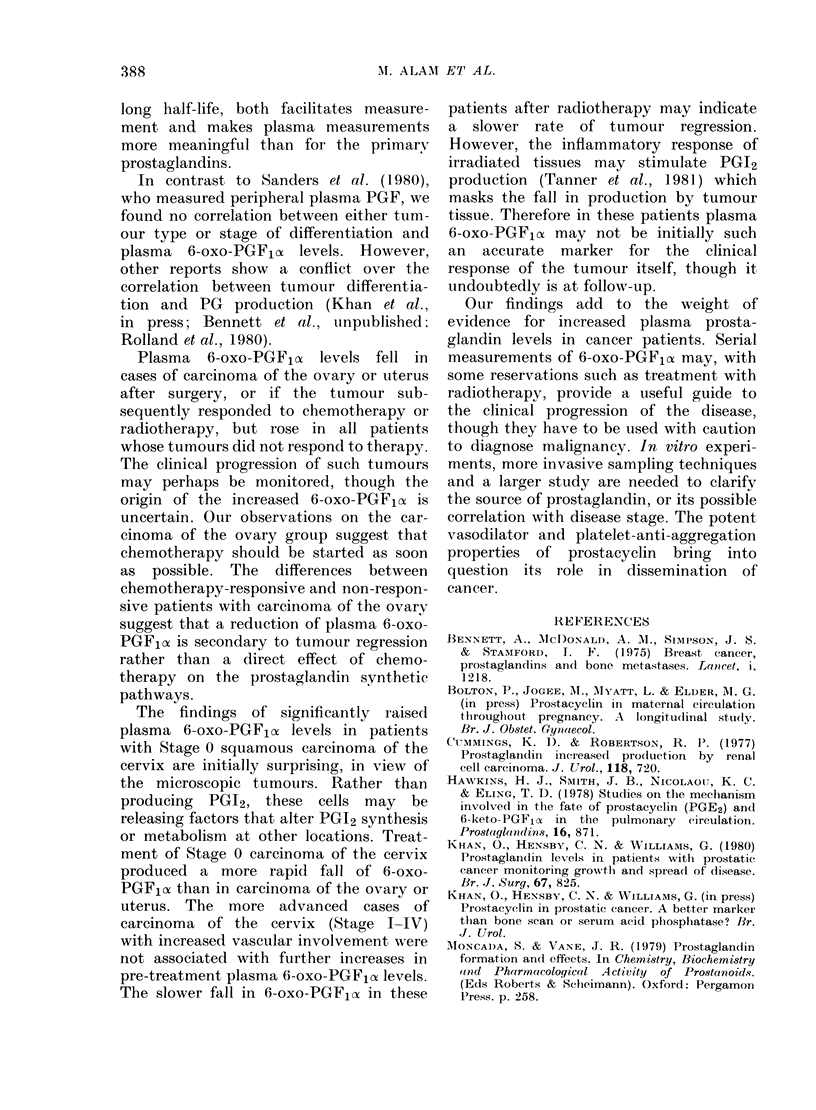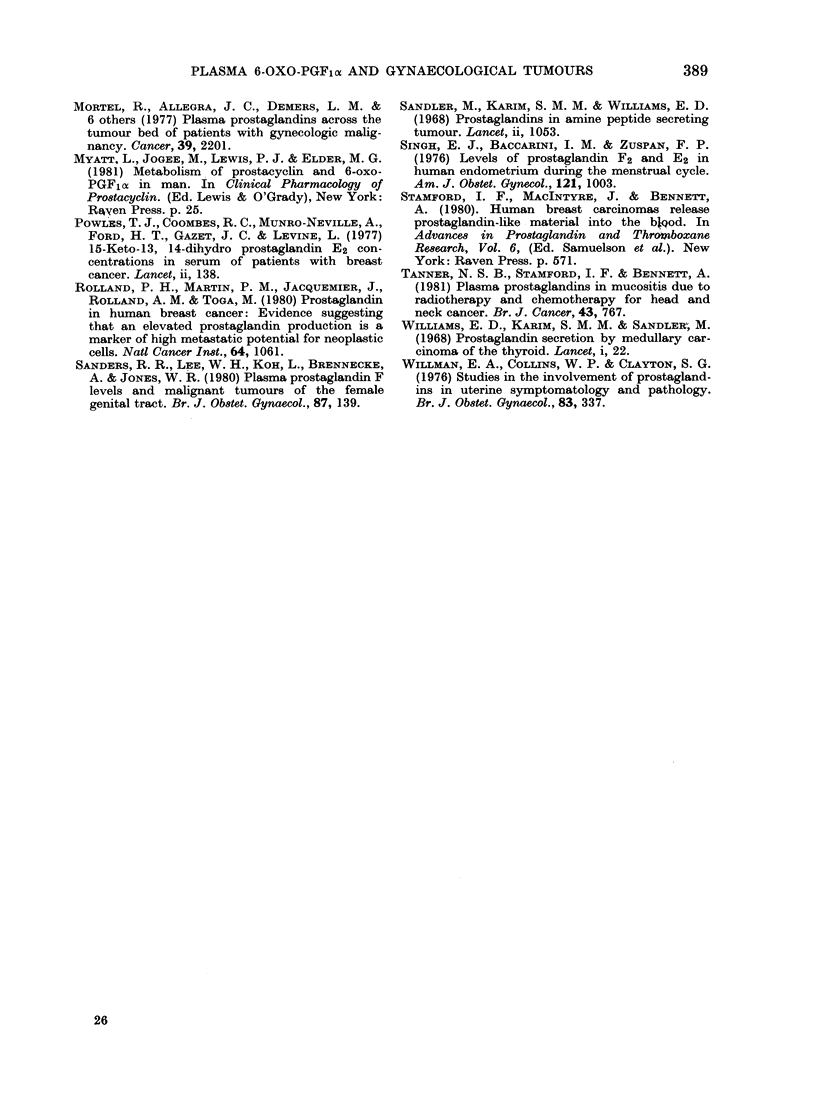# Peripheral plasma immunoreactive 6-oxo-prostaglandin F1 alpha and gynaecological tumours.

**DOI:** 10.1038/bjc.1982.65

**Published:** 1982-03

**Authors:** M. Alam, M. Jogee, W. G. MacGregor, J. W. Dowdell, M. G. Elder, L. Myatt

## Abstract

Peripheral plasma levels of immunoreactive 6-oxo-PGF1 alpha, the stable hydrolysis product of prostacyclin, were significantly higher in female patients with tumours of the genital tract than in normal controls. In the groups with malignant tumours, these high levels declined after operation and/or radiotherapy if the tumour responded to treatment. In patients who did not respond to treatment or with tumour recurrence, levels of plasma 6-oxo-PGF1 alpha remained high or even rose further. Benign gynaecological tumours were also associated with significantly raised plasma 6-oxo-PGF1 alpha levels, and these fell to normal levels immediately on surgical removal of the tumour. Possible reasons for these alterations are described. Further investigations are warranted to see whether serial measurements of plasma 6-oxo-PGF1 alpha could be used as a prognostic index for the clinical status of patients with gynaecological tumours.


*Br. J. Cancer* (1982) **45**, 384

### PERIPHERAL PLASMA IMMUNOREACTIVE 6-OXO-POSTAGLANDIN $F_{1}\alpha$ AND GYNAECOLOGICAL TUMOURS

M. ALAM, M. JOGEE, W. G. MACGREGOR, J. W. DOWDELL,
M. G. ELDER AND L. MYATT

*From the Institute of Obstetrics and Gynaecology, Hammersmith Hospital, Du Cane Road, London W12 0HS*

Received 14 September 1981 Accepted 3 November 1981

**Summary.—Peripheral plasma levels of immunoreactive 6-oxo-PGF$_{1}\alpha$, the stable hydrolysis product of prostacyclin, were significantly higher in female patients with tumours of the genital tract than in normal controls. In the groups with malignant tumours, these high levels declined after operation and/or radiotherapy if the tumour responded to treatment. In patients who did not respond to treatment or with tumour recurrence, levels of plasma 6-oxo-PGF$_{1}\alpha$ remained high or even rose further. Benign gynaecological tumours were also associated with significantly raised plasma 6-oxo-PGF$_{1}\alpha$ levels, and these fell to normal levels immediately on surgical removal of the tumour. Possible reasons for these alterations are described. Further investigations are warranted to see whether serial measurements of plasma 6-oxo-PGF$_{1}\alpha$ could be used as a prognostic index for the clinical status of patients with gynaecological tumours.**

THE PRESENCE of prostaglandins in virtually all cell types so far studied and their potent biological activity has prompted their study in patients with malignant tumours. Increased synthesis of prostaglandins by tumour tissues of thyroid (Williams *et al.*, 1968), phaeochromocytoma and bronchus (Sandler *et al.*, 1968), kidney (Cummings & Robertson, 1977), breast (Bennett *et al.*, 1975) and endometrium (Singh *et al.*, 1976; Willman *et al.*, 1976) have been described. Raised plasma levels of prostaglandins or their metabolites have been measured in the peripheral circulation and across the tumour bed in patients with both genital-tract and breast tumours (Sanders *et al.*, 1980; Powles *et al.*, 1977; Stamford *et al.*, 1980, derived from Mortel *et al.*, 1977). This has led to the suggestion that plasma prostaglandin measurements might be a potentially useful tumour marker.

Prostacyclin ($PGI_2$), a very potent vasodilator and anti-platelet-aggregating agent, is the major product of the cyclo-oxygenase enzyme pathway in a variety of tissues (Moncada & Vane, 1979). Unlike the primary prostaglandins, $PGI_2$ is neither metabolized by the lung (Hawkins *et al.*, 1978) nor is it formed *in vitro* during blood sampling and preparation. Prostacyclin is spontaneously hydrolysed to 6-oxo-PGF$_{1}\alpha$, a metabolite which has been used extensively as a measure of $PGI_2$ production. Khan *et al.* (1980) have found significantly raised levels of plasma 6-oxo-PGF$_{1}\alpha$ in patients with malignant prostatic disease. In this preliminary study we have measured plasma 6-oxo-PGF$_{1}\alpha$ in patients with a variety of gynaecological tumours, and examined serial changes after surgery and/or chemotherapy or radiotherapy and at short-term follow-up.

### PATIENTS AND METHODS

Twenty patients formed the control group. Ten of these underwent major gynaecological surgery for conditions other than a tumour, and 10 were being treated medically for minor problems such as vaginitis.

Of the 52 patients with gynaecological tumours, 15 had benign tumours and 37 malignant tumours. Of the benign-tumour group 12 had uterine fibroids and 3 had an ovarian cyst. The malignant group consisted of carcinoma of the ovary (17), carcinoma of the cervix (16) and carcinoma of the uterus (4).

Venous blood (5 ml) was taken from the antecubital vein into EDTA and immediately centrifuged at 1500 g for 10 min at 4°C. Plasma was separated and stored at −20°C. Levels of 6-oxo-PGF$_{1\alpha}$ were assayed by specific radioimmunoassay (Myatt *et al.*, 1981) within 4 weeks of collection. Collection of blood into indomethacin gave similar values to that collected in EDTA. Therefore, artefactual *in vitro* formation of $PGI_2$ did not influence the results. As radioimmunoassay cannot be entirely specific, the results are expressed as immunoreactive plasma 6-oxo-PGF$_{1\alpha}$ equivalents, and serial changes in immunoreactive 6-oxo-PGF$_{1\alpha}$ were measured. Where applicable, blood samples were collected as follows:

(i) 24 h before operation or start of radiotherapy.
(ii) On the 7th or 8th postoperative day.
(iii) During the 6th or 7th week after surgery or radiotherapy.
(iv) 12 weeks after start of radiotherapy.

All samples were collected between 10:00 and 12:00. None of the patients were given any analgesics known to inhibit prostaglandin synthesis (*e.g.* aspirin and other non steroidal anti-inflammatory drugs).

The statistical differences between mean 6-oxo-PGF$_{1\alpha}$ levels of the various groups was assessed by Wilcoxon's rank sum test or, within the same group, by Wilcoxon's signed rank test for nonparametric statistics.

### RESULTS

The plasma immunoreactive 6-oxo-PGF$_{1\alpha}$ levels in the various study groups are shown in Tables I & II.

### *Control group*

Plasma 6-oxo-PGF$_{1\alpha}$ levels of $93 \pm 32$ pg/ml (mean ± s.d.) in the control group were similar to those previously measured in normal subjects (Myatt *et al.*, 1981); the mean postoperative value of $103 \pm 30$ pg/ml found in the 10 patients who underwent major gynaecological surgery for a non-malignant condition was not significantly different from that of the control values.

### *Benign tumour group*

The pre-operative plasma 6-oxo-PGF$_{1\alpha}$ levels were significantly higher ($P < 0{\cdot}05$) in this group than in the control group. After removal of the tumour, high 6-oxo-PGF$_{1\alpha}$ levels fell to the normal range in all the patients (Fig. 1). With both fibroids and ovarian cysts there appeared to be a direct relationship between the size of the tumour as assessed clinically and the increase in plasma 6-oxo-PGF$_{1\alpha}$ levels.

### *Malignant tumour group*

*Carcinoma of the cervix.*—In 11 patients with Stage 0 carcinoma of the cervix,

TABLE I.—*Plasma immunoreactive 6-oxo-PGF$_{1\alpha}$ in patients with gynaecological tumours*

| Tumour type | Median age (range) | Mean ± s.d. plasma 6-oxo-PGF$_{1\alpha}$ equivalents (pg/ml) | | | | |
|---|---|---|---|---|---|---|
| | | Pre-op | | Post-op (post therapy) | | Follow-up |
| Normal | 39 (56) | $93 \pm 32$ | | $103 \pm 30$ | | |
| Non-malignant (n = 15) | 42 (31) | $181 \pm 120$* | ** | $114 \pm 64$ | | $91 \pm 31$ (n = 10) |
| Fibroids (n = 12) | | $181 \pm 136$ | * | $109 \pm 71$ | | $88 \pm 30$ (n = 9) |
| Ovarian cyst (n = 3) | | $183 \pm 29$ | | $132 \pm 13$ | | |
| Ca cervix (n = 16) | 45 (40) | $215 \pm 103$** | ** | $122 \pm 59$ | ** | $91 \pm 44$ (n = 13) |
| Stage 0 (n = 11) | | $228 \pm 87$** | ** | $113 \pm 56$ | * | $94 \pm 47$ |
| Stage I–IV (n = 5) | | $187 \pm 129$ | | $142 \pm 60$ | | $80 \pm 28$ |

Postoperative samples were taken 7–8 days after operation and follow-up samples at 6–7 weeks. Cancer of the cervix (Stage I–IV) samples were taken at diagnosis, 6 weeks after start of radiotherapy and at 12 weeks. Significance of differences * $P < 0{\cdot}05$, ** $P < 0{\cdot}01$, *** $P < 0{\cdot}001$. Differences between columns are by Wilcoxon signed rank test and $P$ values attached to means refer to the difference from the normal pre-operative value evaluated by the Wilcoxon rank sum test for nonparametric analysis.

TABLE II.—*Plasma immunoreactive 6-oxo-PGF$_{1}\alpha$ in patients with carcinoma of the ovary and uterus*

| Outcome | Median age (range) | Mean ± s.d. plasma 6-oxo-PGF$_{1}\alpha$ equivalents (pg/ml) Pre-op | | Post-op | | Follow-up |
|---|---|---|---|---|---|---|
| (a) Responders (n = 15) | 57·5 (40) | 267 ± 55** | ** | 199 ± 38** | ** | 141 ± 72 (n = 10) |
| (b) Non-responders (n = 6) | 71·5 (15) | 202 ± 103** | | 175 ± 123 | | 225 ± 66*** |

Postoperative samples were taken at 7–8 days after operation and follow-up samples at 6–7 weeks. Significance of differences * $P < 0·05$, ** $P < 0·01$, *** $P < 0·001$, as in Table I.

plasma 6-oxo-PGF$_{1}\alpha$ levels were significantly raised ($P < 0·01$) at the time of diagnosis (Table I). After a total hysterectomy and during subsequent follow-up, plasma 6-oxo-PGF$_{1}\alpha$ levels were comparable to those of controls. The 5 patients with Stage I–IV disease had raised levels of plasma 6-oxo-PGF$_{1}\alpha$ but unrelated to Stage. The rise was not statistically significant, perhaps due to the small number of patients. After radiotherapy all these patients improved clinically, whilst their plasma 6-oxo-PGF$_{1}\alpha$ levels had fallen slightly by 6 weeks, and to the normal range by 3 months (80 ± 28 pg/ml: Table I).

FIG. 1.—Plasma 6-oxo-PGF$_{1}\alpha$ in patients with benign gynaecological tumours. ———, uterine fibroids; – – –, ovarian cysts.

*Carcinoma of the ovary and uterus.*—There were 17 patients with carcinoma of the ovary in this group, with disease ranging from Stage Ia to IV (FIGO), and 4 with carcinoma of the uterus.

The changes in plasma 6-oxo-PGF$_{1}\alpha$ levels in both groups of patients were dependent on the clinical outcome. In view of the few carcinomas of the uterus, both groups were combined for analysis. Pre-operative plasma 6-oxo-PGF$_{1}\alpha$ levels were significantly high, both those responsive and those irresponsive to therapy (Table II) but with no significant difference between these two groups.

### *Responders*

In 12 patients with carcinoma of the ovary the operation was total abdominal hysterectomy, bilateral salpingo-oophorectomy, removal of tumour and omentectomy, followed by chemotherapy. All 12 responded to treatment and are alive, and their 6-oxo-PGF$_{1}\alpha$ levels had fallen considerably after surgery and at follow-up (Fig. 2). Of the 7 cases available for follow-up, 3 were studied more closely. In these patients plasma 6-oxo-PGF$_{1}\alpha$ levels fell postoperatively (Fig. 2) but a subsequent rise was seen while they were awaiting chemotherapy. Once chemotherapy was started, plasma 6-oxo-PGF$_{1}\alpha$ levels fell, with concurrent clinical improvement in all patients.

Three of the 4 patients with carcinoma of the uterus were operable, responded to radiotherapy, and are alive and well. Both postoperatively and after radiotherapy, plasma 6-oxo-PGF$_{1\alpha}$ levels fell to normal. In the combined group of responsive patients, significant reductions of plasma 6-oxo-PGF$_{1\alpha}$ occurred in the postoperative and follow-up periods (Table II). Although the levels were still significantly higher than normal postoperatively, they had fallen to within the normal range at follow-up.

FIG. 2.—Plasma 6-oxo-PGF$_{1\alpha}$ in patients with carcinoma of the ovary. ———, responders; – – –, non-responders; *, chemotherapy; ◆, radiotherapy.

FIG. 3.—Plasma 6-oxo-PGF$_{1\alpha}$ in patients with carcinoma of the cervix. ———, Stage 0; – – –, Stages I–IV.

*Non-responders*

Five patients with carcinoma of the ovary and one with carcinoma of the uterus were found to be inoperable. None of these responded to chemotherapy and all subsequently died. The mean plasma 6-oxo-PGF$_{1\alpha}$ levels were significantly raised before laparotomy (Table II), were not significantly affected by chemotherapy, and were still significantly above normal at follow-up. This contrasted with the group of patients who responded to therapy, in all of whom plasma 6-oxo-PGF$_{1\alpha}$ fell.

### DISCUSSION

Plasma 6-oxo-PGF$_{1\alpha}$ levels in the control group of patients were not altered by major surgery. To our knowledge no other study has shown an increase in prostaglandin production associated with a benign tumour. The apparent relationship between benign tumour size (fibroid or ovarian cyst) and plasma 6-oxo-PGF$_{1\alpha}$ suggested either that the tumour produced and released $PGI_2$ into the peripheral circulation or that increased $PGI_2$ production may be associated with the increased vascular supply to these tumours. Plasma 6-oxo-PGF$_{1\alpha}$ levels rise during normal pregnancy, perhaps in association with the increased uterine vascularity (Bolton *et al.*, in press).

Primary prostaglandin concentrations are increased in plasma (Sanders *et al.*, 1980), tumour tissues (Singh *et al.*, 1976; Willman *et al.*, 1976) and across the tumour bed (Stamford *et al.*, 1980, derived from Mortel *et al.*, 1977) in patients with gynaecological tumours. The lack of $PGI_2$ metabolism by the lung (Hawkins *et al.*, 1978), and its spontaneous hydrolysis in plasma to 6-oxo-PGF$_{1\alpha}$, which has a relatively long half-life, both facilitates measurement and makes plasma measurements more meaningful than for the primary prostaglandins.

In contrast to Sanders *et al.* (1980), who measured peripheral plasma PGF, we found no correlation between either tumour type or stage of differentiation and plasma 6-oxo-$PGF_{1}\alpha$ levels. However, other reports show a conflict over the correlation between tumour differentiation and PG production (Khan *et al.*, in press; Bennett *et al.*, unpublished; Rolland *et al.*, 1980).

Plasma 6-oxo-$PGF_{1}\alpha$ levels fell in cases of carcinoma of the ovary or uterus after surgery, or if the tumour subsequently responded to chemotherapy or radiotherapy, but rose in all patients whose tumours did not respond to therapy. The clinical progression of such tumours may perhaps be monitored, though the origin of the increased 6-oxo-$PGF_{1}\alpha$ is uncertain. Our observations on the carcinoma of the ovary group suggest that chemotherapy should be started as soon as possible. The differences between chemotherapy-responsive and non-responsive patients with carcinoma of the ovary suggest that a reduction of plasma 6-oxo-$PGF_{1}\alpha$ is secondary to tumour regression rather than a direct effect of chemotherapy on the prostaglandin synthetic pathways.

The findings of significantly raised plasma 6-oxo-$PGF_{1}\alpha$ levels in patients with Stage 0 squamous carcinoma of the cervix are initially surprising, in view of the microscopic tumours. Rather than producing $PGI_2$, these cells may be releasing factors that alter $PGI_2$ synthesis or metabolism at other locations. Treatment of Stage 0 carcinoma of the cervix produced a more rapid fall of 6-oxo-$PGF_{1}\alpha$ than in carcinoma of the ovary or uterus. The more advanced cases of carcinoma of the cervix (Stage I–IV) with increased vascular involvement were not associated with further increases in pre-treatment plasma 6-oxo-$PGF_{1}\alpha$ levels. The slower fall in 6-oxo-$PGF_{1}\alpha$ in these patients after radiotherapy may indicate a slower rate of tumour regression. However, the inflammatory response of irradiated tissues may stimulate $PGI_2$ production (Tanner *et al.*, 1981) which masks the fall in production by tumour tissue. Therefore in these patients plasma 6-oxo-$PGF_{1}\alpha$ may not be initially such an accurate marker for the clinical response of the tumour itself, though it undoubtedly is at follow-up.

Our findings add to the weight of evidence for increased plasma prostaglandin levels in cancer patients. Serial measurements of 6-oxo-$PGF_{1}\alpha$ may, with some reservations such as treatment with radiotherapy, provide a useful guide to the clinical progression of the disease, though they have to be used with caution to diagnose malignancy. *In vitro* experiments, more invasive sampling techniques and a larger study are needed to clarify the source of prostaglandin, or its possible correlation with disease stage. The potent vasodilator and platelet-anti-aggregation properties of prostacyclin bring into question its role in dissemination of cancer.

### REFERENCES

BENNETT, A., McDONALD, A. M., SIMPSON, J. S. & STAMFORD, I. F. (1975) Breast cancer, prostaglandins and bone metastases. *Lancet*, i, 1218.

BOLTON, P., JOGEE, M., MYATT, L. & ELDER, M. G. (in press) Prostacyclin in maternal circulation throughout pregnancy. A longitudinal study. *Br. J. Obstet. Gynaecol.*

CUMMINGS, K. D. & ROBERTSON, R. P. (1977) Prostaglandin increased production by renal cell carcinoma. *J. Urol.*, **118**, 720.

HAWKINS, H. J., SMITH, J. B., NICOLAOU, K. C. & ELING, T. D. (1978) Studies on the mechanism involved in the fate of prostacyclin ($PGE_2$) and 6-keto-$PGF_{1}\alpha$ in the pulmonary circulation. *Prostaglandins*, **16**, 871.

KHAN, O., HENSBY, C. N. & WILLIAMS, G. (1980) Prostaglandin levels in patients with prostatic cancer monitoring growth and spread of disease. *Br. J. Surg*, **67**, 825.

KHAN, O., HENSBY, C. N. & WILLIAMS, G. (in press) Prostacyclin in prostatic cancer. A better marker than bone scan or serum acid phosphatase? *Br. J. Urol.*

MONCADA, S. & VANE, J. R. (1979) Prostaglandin formation and effects. In *Chemistry, Biochemistry and Pharmacological Activity of Prostanoids.* (Eds Roberts & Scheimann). Oxford: Pergamon Press. p. 258.

MORTEL, R., ALLEGRA, J. C., DEMERS, L. M. & 6 others (1977) Plasma prostaglandins across the tumour bed of patients with gynecologic malignancy. *Cancer*, **39**, 2201.

MYATT, L., JOGEE, M., LEWIS, P. J. & ELDER, M. G. (1981) Metabolism of prostacyclin and 6-oxo-$PGF_{1\alpha}$ in man. In *Clinical Pharmacology of Prostacyclin*. (Ed. Lewis & O'Grady), New York: Raven Press. p. 25.

POWLES, T. J., COOMBES, R. C., MUNRO-NEVILLE, A., FORD, H. T., GAZET, J. C. & LEVINE, L. (1977) 15-Keto-13, 14-dihydro prostaglandin $E_2$ concentrations in serum of patients with breast cancer. *Lancet*, ii, 138.

ROLLAND, P. H., MARTIN, P. M., JACQUEMIER, J., ROLLAND, A. M. & TOGA, M. (1980) Prostaglandin in human breast cancer: Evidence suggesting that an elevated prostaglandin production is a marker of high metastatic potential for neoplastic cells. *Natl Cancer Inst.*, **64**, 1061.

SANDERS, R. R., LEE, W. H., KOH, L., BRENNECKE, A. & JONES, W. R. (1980) Plasma prostaglandin F levels and malignant tumours of the female genital tract. *Br. J. Obstet. Gynaecol.*, **87**, 139.

SANDLER, M., KARIM, S. M. M. & WILLIAMS, E. D. (1968) Prostaglandins in amine peptide secreting tumour. *Lancet*, ii, 1053.

SINGH, E. J., BACCARINI, I. M. & ZUSPAN, F. P. (1976) Levels of prostaglandin $F_2$ and $E_2$ in human endometrium during the menstrual cycle. *Am. J. Obstet. Gynecol.*, **121**, 1003.

STAMFORD, I. F., MACINTYRE, J. & BENNETT, A. (1980). Human breast carcinomas release prostaglandin-like material into the blood. In *Advances in Prostaglandin and Thromboxane Research, Vol. 6*, (Ed. Samuelson *et al.*). New York: Raven Press. p. 571.

TANNER, N. S. B., STAMFORD, I. F. & BENNETT, A. (1981) Plasma prostaglandins in mucositis due to radiotherapy and chemotherapy for head and neck cancer. *Br. J. Cancer*, **43**, 767.

WILLIAMS, E. D., KARIM, S. M. M. & SANDLER, M. (1968) Prostaglandin secretion by medullary carcinoma of the thyroid. *Lancet*, i, 22.

WILLMAN, E. A., COLLINS, W. P. & CLAYTON, S. G. (1976) Studies in the involvement of prostaglandins in uterine symptomatology and pathology. *Br. J. Obstet. Gynaecol.*, **83**, 337.